# The effects of 17 alpha-estradiol to inhibit inflammation in vitro

**DOI:** 10.1186/s13293-017-0151-9

**Published:** 2017-09-06

**Authors:** Roberta S. Santos, Luciana A. de Fatima, Aaron P. Frank, Everardo M. Carneiro, Deborah J. Clegg

**Affiliations:** 1 Biomedical Sciences Dept, Diabetes and Obesity Research Division, Cedars-Sinai Medical Center, 8700 Beverly Blvd, Los Angeles, CA 90048 USA; 2 Obesity and Comorbidities Research Center (OCRC), Institute of Biology, State University of Campinas-UNICAMP, Campinas, SP Brazil

**Keywords:** 17 Alpha-estradiol (17 α-E2), 17 Beta-estradiol (17 β-E2), Inflammation, Cell culture, Sexual dimorphism

## Abstract

**Background:**

17 Alpha-estradiol (17 α-E2) is a natural, non-feminizing stereoisomer of 17 beta-estradiol (17 β-E2). Whereas much is known about the physiological effects of 17 β-E2, much less is known about 17 α-E2. For example, 17 β-E2 exerts anti-inflammatory effects in neurons and adipocytes through binding and activation of estrogen receptor alpha (ERα); however, if 17 α-E2 has similar effects on inflammation is currently unknown.

**Methods:**

To begin to address this, we analyzed the ability of 17 α-E2 and 17 β-E2 to suppress lipopolysaccharide (LPS)-induced inflammation in vitro using embryonic fibroblast cells (MEF) from wild type and total body ERα (ERKO) male and female mice. Additionally, we further probed if there were sex differences with respect to the effects of E2s using primary pre-adipocyte cells from C57BL/6J male and female mice. Also, we probed mechanistically the effects of E2s in fully differentiated 3T3-L1 cells.

**Results:**

Both E2s decreased LPS-induced markers of inflammation *Tnf-α* and *Il-6*, and increased the anti-inflammatory markers *Il-4* and IL-6 receptor (*Il-6ra*) in MEF cells. To begin to understand the mechanisms by which both E2’s mediate their anti-inflammatory effects, we probed the role of ERα using two methods. First, we used MEF cells from ERKO mice and found reductions in ERα diminished the ability of 17 α-E2 to suppress *Tnf-α* in female but not in male cells, demonstrating a sexual dimorphism in regard to the role of ERα to mediate 17 α-E2’s effects. Second, we selectively reduced the expression of ERα in 3T3-L1 cells using siRNA and found reductions in ERα diminished the ability of both E2s to suppress *Tnf-α* and *Il-6* expression. Lastly, to determine the mechanisms by which E2s reduce inflammation, we explored the role of NFκB-p65 and found both E2s decreased NFκB-p65 expression.

**Conclusions:**

In conclusion, we demonstrate for the first time that 17 α-E2, as well as 17 β-E2, suppresses inflammation through their effects on ERα and NFκB-p65.

**Electronic supplementary material:**

The online version of this article (10.1186/s13293-017-0151-9) contains supplementary material, which is available to authorized users.

## Background

Estrogens play a crucial role in suppressing the development of chronic inflammatory diseases [1] and mitigating the impact of inflammation that occurs in diseases such as obesity and type II diabetes mellitus [2]. There is a wealth of information indicating that adipose tissues become inflamed in obesity, and adipose tissue inflammation is highly correlated with insulin resistance, cancer, and other metabolic sequelae associated with increased adiposity [3, 4]. Therefore, preventing or treating adipose tissue inflammation is undoubtedly important to prevent the development and slow the progression of metabolic diseases. Importantly, there appears to be a sexual dimorphism regarding the prevalence of diseases such as obesity, type II diabetes, and cardiovascular disease, with adult males presenting with a higher prevalence and enhanced disease risk when compared to adult females; however, following menopause, women become as susceptible as men to diseases associated with obesity [2]. Specifically, it has been demonstrated that loss of estrogens during the menopausal transition leads to increased expression and secretion of pro-inflammatory cytokines such as tumor necrosis factor-alpha (TNF-α) and interleukin 6 (IL-6) [5].

Estrogens are a family of compounds that include 17 alpha-estradiol (17 α-E2) as well as 17 beta-estradiol (17 β-E2). 17 β-E2 is the main circulating and biologically most active form of estrogen [6, 7], and it is well established that 17 β-E2 decreases markers of inflammation [8]; however, the exact mechanisms by which it interferes with inflammatory processes are not fully understood. It appears that most of the anti-inflammatory effects of estrogens in non-reproductive tissues occur by activating estrogen receptors (ERs), mainly ERalpha (ERα) [9]. We have previously demonstrated that ERα is the predominant ER in the adipose tissue, and there are studies suggesting that ERα polymorphisms lead to adipose tissue accumulation, increased insulin resistance, and inflammation [10]. It has been shown that 17 β-E2 binds ERα and inhibits the transcription of *Tnf*-α and *Il-6* genes [11, 12]. In addition, through a transcriptional cross-talk between ERα and NFkappaB (NFκB), 17 β-E2 inhibits the transcription of *Il-6* [13]. Furthermore, there are data to suggest that 17 β-E2, in a nongenomic manner, can inhibit inflammation by modulating NFκB trafficking to the nucleus through activation of the PI3K/AKT pathway [14].

17 α-E2 is a natural, non-feminizing isomer of 17 β-E2 [15], and its mechanisms of action and function are relatively unknown. 17 α-E2 has been found in various tissues of adult male and female mice. Its concentrations are higher in the brain and adrenal glands compared to 17 β-E2 and lower in the ovaries, uterus, and serum [16]. Currently, there are less data on the location and tissue content of 17 α-E2 in humans; however, there is evidence of endogenous 17 α-E2 in the urine and serum of humans [17, 18]. Importantly, 17 α-E2 is different from the synthetic compound mostly used in oral contraceptives and postmenopausal hormone therapy, 17 α-ethynylestradiol [19, 20]; importantly, little is known about either of these estrogenic compounds and even less is known about their anti-inflammatory activities [21–24]. Recently, aged male mice fed a diet enriched in 17 α-E2 had reductions in body adiposity and markers of inflammation in plasma and adipose tissue [20], and there are data to suggest that 17 α-E2 may have neuroprotective effects [25]. 17 α-E2 appears to have the capacity to bind to ERs, but with lower affinity than 17 β-E2 [26].

In contrast to 17 α-E2, 17 β-E2 also has feminizing effects and can lead to uterine and ER-positive breast cancer in postmenopausal women [27–30]. Recently, Stout et al. (2017) [20] demonstrated reductions in inflammatory markers in adipose tissue in mice who were fed diets enriched in 17 α-E2. Since adipose tissue inflammation is key to the development of several diseases related to obesity, 17 α-E2 may be useful in treating metabolic disease, while avoiding the side effects of traditional 17 β-E2 exposure. Here, we explored the anti-inflammatory effects of both E2s in vitro to begin to characterize the mechanisms which underlie the potential therapeutic benefit of 17 α-E2 for women and, possibly, men.

## Methods

### Cell culture

Briefly, mouse embryonic fibroblast (MEF) cells were generated from C57BL/6J WT and total body ERα knockout (ERKO) mice. ERKO mice were a gift from Pierre Chambon [31]. Embryos from WT and ERKO mice were harvested from two approximately 2–3-month-old dams, who were fed a chow diet optimized for breeding (#2019, Harlan Teklad, Madison, WI). Mice were mated and conception was visually confirmed by the presence of a mating plug. Thirteen days after conception, dams were euthanized and embryos removed. Embryos were sacrificed by decapitation, and embryo heads were collected for genotyping. The embryos were then washed in sterile PBS, minced with a razor blade in 2 mL trypsin (025%, Gibco). Following 5 min of incubation at 37 °C, the minced tissue was pipetted up and down to produce a homogenous cell suspension. The homogenate was then incubated an additional 5 min at 37 °C and then evenly applied to a 50 mL petri dish containing 25 mL of culture media (DMEM High glucose medium with 10% FBS, 1% Pen/Strep, and 1 mM NaPyr) and incubated at 37 °C. Cells were passaged once they reached ~ 90% confluency. Primary pre-adipocytes were generated as previously described [32].

MEF cells were cultured in DMEM no-phenol red (Thermo Fisher Scientific, Walhman, USA), supplemented with 10% charcoal: dextran stripped fetal bovine serum (FBS, Gemini Bio Products, Sacramento, USA), 1% pen-strep 10,000 U/ml (Thermo Fisher), and 1% sodium pyruvate 100 mM (Thermo Fisher). Primary pre-adipocytes were cultured in DMEM/F-12 with GlutaMAX phenol red (Thermo Fisher) supplemented with 10% FBS and 1% pen-strep 10,000 U/ml. 3T3-L1 cells were grown in DMEM phenol-red media (ATCC, Manassas, USA), supplemented with 10% Bovine calf bovine serum (ATCC) and 1% pen-strep. After 48 h of confluency, DMEM was supplemented with methylisobutylxanthine (IBMX, 0.5 mM, Sigma, St Louis, USA), dexamethasone (1 μM, BD Biosciences, San Jose, USA), and insulin 10 μg/ml (Sigma) [33], and serum was replaced FBS. Following 48 h of exposure, cells were cultured in DMEM supplemented only with insulin. For all cells, medium was replaced by DMEM no phenol-red supplemented with 10% charcoal: dextran stripped FBS 24 h before and during treatments.

Cells were treated for the indicated time with 17 α-E2 (Sigma) or 17 β-E2 (Sigma) at 10 μM concentration. Inflammation was induced by LPS at a concentration of 10 ng/ml (Sigma) either alone or in combination with the respective estrogen.

### RNAi in cell culture

In this series of experiments, we used siRNA to knockdown *Esr-1* (ERα gene) in the presence and absence of estrogens to determine if ERα mediates estrogens’ effects on inflammation. To do this, 3T3-L1 cells were cultured in 12-well plates and transfected at day 10 of differentiation with siRNA targeting murine *Esr-1* (siGENOME Mouse si*Esr1*, GE Dharmacon, Lafayette, USA, at the concentration of 100 nM). As a control, cells were treated with an unrelated control/scrambled sequence siRNA at the same concentration (siGENOME non-targeting siRNA, GE Dharmacon). Lipofectamine RNAi max was used as the transfection reagent (Thermo Fisher, at the concentration of 20 рM/μl), according to the manufacturer’s instructions. Seventy-two hours after siRNA transfection, cells were treated with estrogens and LPS as indicated, and then lysed for RNA extraction.

### Quantitative real-time PCR

Relative expression of the pro-inflammatory genes Tumor Necrosis Factor Alpha (*Tnf-α*); interleukin 6 (*Il-6)*; and Nuclear Factor Kappa B Subunit 1 (*Nfκb1*), and anti-inflammatory genes interleukin 4 (*Il-4)*, and interleukin 6 receptor alpha (*Il-6rα)* were quantified following LPS and/or E2s using qPCR. To determine the impact of treatments on ERα, we also quantified *Esr-1* gene expression. Cells were washed twice with PBS (Thermo Fisher) and lysed in 500 μl of TRIzol® reagent (Thermo Fisher). RNA from cells was extracted and isolated using the RNeasy kit (Qiagen, Germantown, USA), according to the manufacturer’s instructions. The concentration and purity of RNA were determined by spectrophotometric analysis (NanoDrop ND-1000, Thermo Fisher), and all samples had a A_260_/A_280_ ratio around 2.0 [34]. Total cDNA was synthesized using the High Capacity cDNA Reverse Transcription kit (Applied Biosystems, Foster City, USA). Real-time qPCR was performed using Taqman Universal Mastermix II (Applied Biosystems) and Taqman specific primers, on the QuantStudio 12 K Flex Real-Time PCR System. Mouse *Gapdh* was used as reference gene, and data was normalized and relative expression determined using 2^-∆∆ CT^ method.

### Western blotting

We determined total protein levels of ERα, as well as protein levels and cellular location of NFκB p65. NFκB fractions in the cytosol or nucleus were normalized to total NFκB expression. Briefly, for total protein, 150 μl of RIPA buffer (Thermo Fisher), supplemented with anti-phosphatase cocktail (PhosphoSTOP Easy pack, Sigma) and anti-protease cocktail (Complete mini protease inhibitor, Sigma), was used per well. After sample centrifugation, the supernatant was separated and used to quantify protein concentration and prepare samples for gel electrophoresis and Western blotting. For cell fractionation, the protocol adapted from Silva et al. (2005) [35] was used.

Protein concentrations were determined by BCA (Thermo Fisher). Proteins (30–50 μg) were separated by electrophoresis (Bio-Rad, Hercules, USA) and electro-transferred to nitrocellulose membrane (Trans-turbo transfer pack, Bio-Rad). Non-specific binding sites were saturated by incubation of membranes for 1 h in TBS (supplemented with 1% Tween 20) and 5% non-fat powdered milk, followed by overnight incubation with primary antibodies (NFκB p65, Sc8008, St Cruz Biotechnology, Dallas, USA; ERα, Sc542, St Cruz, at the concentration of 1:1000). Appropriate secondary antibodies were used (Goat anti-mouse, SeraCare Life Sciences, Milford, USA, or Goat anti-rabbit, SeraCare, at the concentration of 1:10,000), followed by enhanced chemiluminescence assay (Bio-Rad). The optical density of blots was analyzed using ImageLab software (Bio-Rad), and β-actin (Sc1615, St Cruz) or histone (Sc10809, St Cruz) were used for normalization. The results were expressed as arbitrary units in comparison to the control group, which was set as 1.0.

### Immunofluorescence

In this series, we verified protein expression and location of NFκB p65 and ERα. To do this, cells were fixed with 4% paraformaldehyde and permeabilized with 0.5% saponin. Cells were stained for primary antibodies NFκB p65 or ERα overnight (at the concentration of 1:50), and then incubated with fluorescent-labeled secondary antibody (Goat to mouse-Alexa Fluor, ab150116, Abcam, Cambridge, USA; Goat to rabbit- FITC, ab6717, Abcam, at the concentration of 1:200), followed by DAPI incubation. Slides were mounted with Vectashield (Vector Laboratories, Burlingame, USA). Images were captured using Keyence® BZ-9000 microscope and analyzed by Keyence® BZ-9000 software.

### Statistical analysis

Data is presented as mean ± SEM of three independent experiments. For primary cells, two mice were pooled and processed for each experiment, which yielded three biological replicates [36, 37]. Statistical analysis was performed with GraphPad Prism version 6.0 for Windows (GraphPad software, San Diego, CA, USA). One-way ANOVA followed by Tukey post-test comparisons and two-tailed paired Student’s test were used as appropriate. *P* < 0.05 was considered statistically significant.

## Results

### 17 α-E2 and 17 β-E2 attenuated LPS-induced inflammatory markers in both male and female MEF cells

**Fig. 1:**
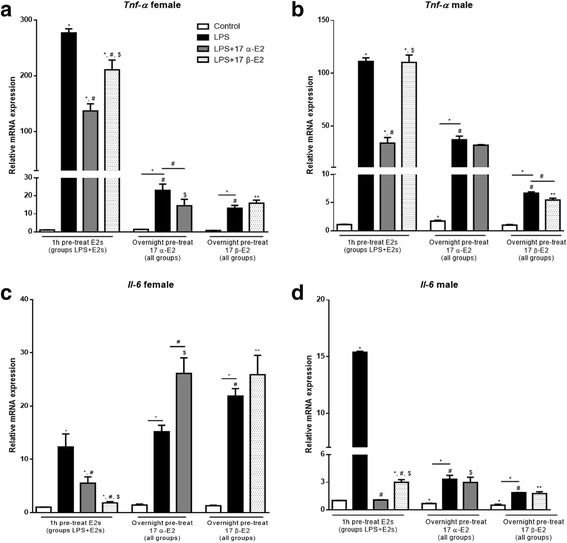
Effects of 17 α-E2 and 17 β-E2 on mouse embryonic fibroblast (MEF) cells derived from C57BL/6J female and male mice. **a** Cells were pre-treated with vehicle (control and LPS groups) or estrogens for 1 h (LPS + 17 α-E2 and LPS + 17 β-E2 groups). Following which, the media was changed to either new media plus LPS (LPS group), or LPS + 17 α-E2 (LPS + 17 α-E2 group), or LPS + 17 β-E2 (LPS + 17 β-E2 group) for 2 h. **b** All groups were pre-treated overnight (approximately 12 h) with 17 α-E2 (control, LPS, and LPS + 17 α-E2 groups). Media was replaced on the next day by new media supplemented with LPS (LPS groups), or LPS + 17 α-E2 (LPS + 17 α-E2 group) for 2 h. **c** All groups were pre-treated overnight (approximately 12 h) with 17 β-E2 (control, LPS, and LPS + 17 β-E2 groups). Media was replaced on the next day by new media supplemented with LPS (LPS groups), or LPS + 17 β-E2 (LPS + 17 β-E2 group) for 2 h. Relative mRNA expression of *Tnf-α* (**a**–**b**), and *Il-6* (**c**–**d**). Data are presented as mean ± SEM values. Asterisk indicates *P* < 0.05 × control group (C). Pound indicates *P* < 0.05 × LPS group. Dollar sign indicates *P* < 0.05 × LPS + 17 α-E2 group. Two asterisks indicates *P* < 0.05 × LPS + 17 β-E2 group (*n* = 3 independent rounds of cells)

To begin to address the role of estrogens (E2s) on inflammatory markers in vitro, we directly applied 17 α-E2 and 17 β-E2 to mouse embryonic fibroblast (MEF) cells derived from male and female C57BL/6J mice. To induce inflammation, MEFs were treated with LPS followed by the two different estrogens. First, to determine if estrogens are able to suppress LPS-induced inflammation, cells were pre-treated overnight (12 h) with estrogens, followed by a 2-h LPS treatment. In an additional series of experiments to determine if estrogens are able to suppress LPS-induced inflammation with shorter exposure, cells were pre-treated with E2s for only 1 h, followed by 2 h LPS treatment. Long pre-exposure to 17 α-E2 or 17 β-E2 prevented LPS-induced inflammation as demonstrated by reductions in *Tnf-α* mRNA expression in both male and female cells; however, following the short-term exposure, only 17 α-E2 was capable of reducing *Tnf-α* in both sexes (Fig. 1a, b). Interestingly, the longer exposure to E2s did not attenuate LPS-induced *Il-6* expression in female cells, but did in male cells; whereas, shorter exposure of both E2s decreased *Il-6* in both female (Fig. 1c) and male cells (Fig. 1d).

**Fig. 2:**
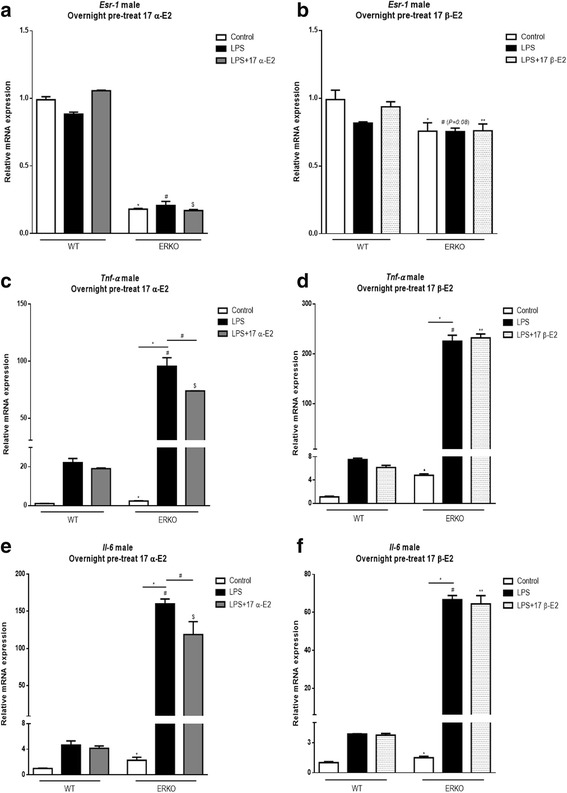
Effects of 17 α-E2 and 17 β-E2 on mouse embryonic fibroblast (MEF) cells derived from C57BL/6J WT and ERKO male mice pre-treated overnight with 17 α-E2 (A, C, and E), or 17 β-E2 (B, D, and E) for 12 h, followed by 2 h LPS. Relative mRNA expression of *Esr-1* (**a**–**b**), *Tnf-α* (**c**–**d**), and *Il-6* (**e**–**f**). Data are presented as mean ± SEM values. Asterisk indicates *P* < 0.05 × control group (C). Pound indicates *P* < 0.05 × LPS group. Dollar sign indicates *P* < 0.05 × LPS + 17 α-E2 group. Two asterisks indicates *P* < 0.05 × LPS + 17 β-E2 group (*n* = 3 independent rounds of cells)

**Fig. 3:**
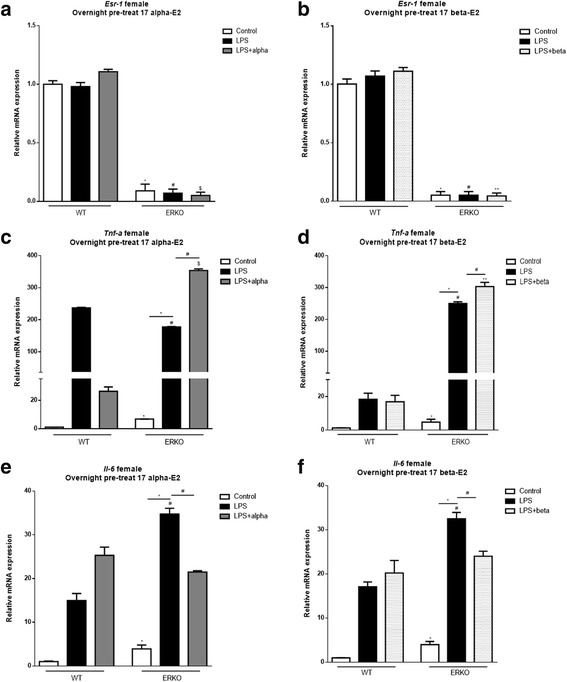
Effects of 17 α-E2 and 17 β-E2 on mouse embryonic fibroblast (MEF) cells derived from C57BL/6J WT and ERKO female mice pre-treated overnight with 17 α-E2 (A, C, and E), or 17 β-E2 (B, D and E) for 12 h, followed by 2 h LPS. Relative mRNA expression of *Esr-1* (**a**–**b**), *Tnf-α* (**c**–**d**), and *Il-6* (**e**–**f**). Data were presented as mean ± SEM values. Asterisk indicates *P* < 0.05 × control group (C). Pound indicates *P* < 0.05 × LPS group. Dollar sign indicates *P* < 0.05 × LPS + 17 α-E2 group. Two asterisks indicates *P* < 0.05 × LPS + 17 β-E2 group (*n* = 3 independent rounds of cells)

To begin to determine the role of ERα to mediate E2s’ effects on inflammation, MEF cells were generated from mice lacking ERα (ERKO) and were tested using a similar paradigm (Fig. 2a, b, 3a, b). ERKO cells at baseline had higher markers of *Tnf-α* and *Il-6* genes both in male (Fig. 2c–f) and female (Fig. 3c–f) cells when compared to wild type, control cells. In male ERKO cells, 17 α-E2 treatment reduced LPS-induced *Tnf-α* and *Il-6* expression, indicating 17 α-E2 might activate other ERs to reduce inflammatory markers in male cells (Fig. 2c–f). In female ERKO cells, 17 α-E2 treatment reduced LPS-induced *Il-6* but not *Tnf-α* expression (Fig. 3c–f), indicating the ability of 17 α-E2 to decrease *Tnf-α* gene expression depends on ERα only in female cells. These intriguing data strongly support the hypothesis that male and female cells differ with respect to their response to 17 α-E2 to modulate inflammation, and ERα might modulate 17 α-E2’s effects only in female cells.

### 17 α-E2 and 17 β-E2 attenuated LPS-induced inflammatory markers in primary pre-adipocytes derived from male and female mice

**Fig. 4:**
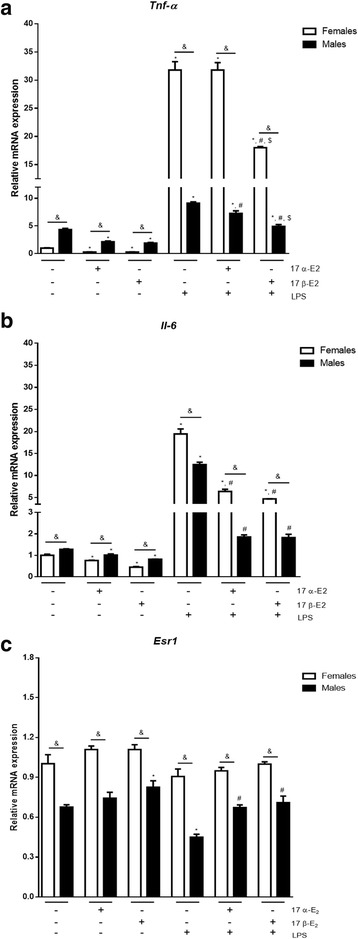
Effects of 17 α-E_2_ and 17 β-E_2_ on modulation of LPS-induced inflammatory markers in primary pre-adipocyte cells derived from C57BL/6J female and male mice. Cells were pre-treated with E2s for 1 h, followed by 2 h LPS treatment. Relative mRNA expression of *Tnf-α* (**a**), *Il-6* (**b**), and *Esr1* (**c**). Data are presented as mean ± SEM values. Asterisk indicates *P* < 0.05 compared to control group (C). Pound indicates *P* < 0.05 × LPS group. Dollar sign indicates *P* < 0.05 × LPS + 17 α-E2 group. Ampersand indicates *P* < 0.05 × females. (*n* = 3 independent rounds of cells)

Since the effects of 17 α-E2 to modulate inflammatory markers were more pronounced in the short-term exposure in MEF cells, we further investigated its effects on primary pre-adipocytes using this protocol. Primary pre-adipocytes were extracted from C57BL/6J male and female mice; at baseline, male cells had higher expression of *Tnf-α* and *Il-6* when compared to females (Fig. 4a, b). 17 β-E2 reduced LPS-induced inflammatory markers in both male and female cells. Following LPS exposure, 17 α-E2 decreased *Tnf-α* expression in male but not in female cells (Fig. 4a). Therefore, in a more developed model of adipose tissues (primary pre-adipocyte cells), 17 α-E2 was able to reduce *Il-6* in cells derived from both sexes, but it decreased *Tnf-α* only in male cells. Male cells presented lower levels of *Esr1* expression than female cells (Fig. 4c); consistent with our previous ERKO MEF data, this suggests 17 α-E2 might activate other ERs to reduce inflammation in males.

### 17 α-E2 and 17 β-E2 attenuated LPS-induced inflammatory markers in differentiated 3T3-L1 adipocytes in an ERα-dependent manner

**Fig. 5:**
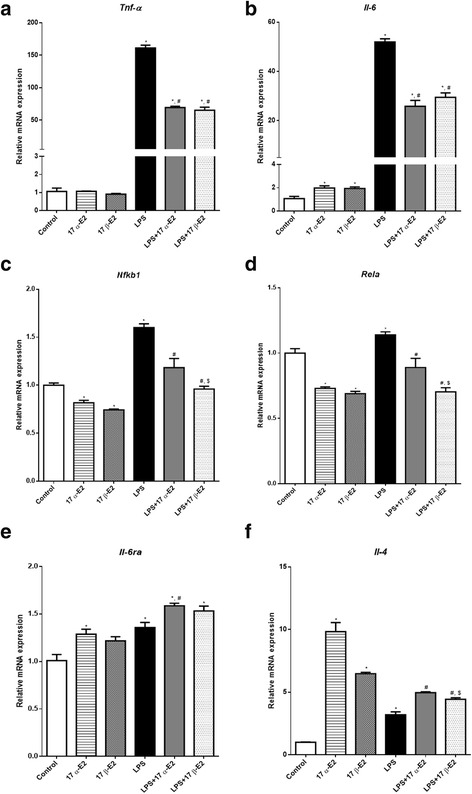
Effects of 17 α-E2 and 17 β-E2 on modulation of inflammatory markers in 3T3-L1 differentiated cells. Relative mRNA expression of *Tnf-α* (**a**), *Il-6* (**b**), *Nfκb1* (**c**), *Rela* (**d**), *Il-6ra* (**e**), and *Il-4* (**f**). Data are presented as mean ± SEM values. Asterisk indicates *P* < 0.05 compared to control group (C). Pound indicates *P* < 0.05 × LPS group. Dollar sign indicates *P* < 0.05 × LPS + 17 α-E2 group. (*n* = 3 independent rounds of cells)

In 3T3-L1 fully differentiated adipocytes, we confirmed that the treatments with LPS and E2s were not altering the viability of the cells (Additional file 1: Figure S1a, b). Consistent with our previous results, LPS treatment increased mRNA expression of *Tnf-α* and *Il-6*, while both E2s decreased LPS-induced *Tnf-α* and *Il-6* expression (Fig. 5a, b). To begin to assess if this was unique to these two inflammatory markers, we also probed for *Nfκb1* and *Rela*, additional inflammatory markers within the NFκB family. Consistently, we found that E2s reduced expression of these markers (Fig. 5c, d). Additionally, both E2s increased *Il-4* and *Il-6ra* mRNA, markers of anti-inflammatory pathway induction (Fig. 5e, f).

**Fig. 6:**
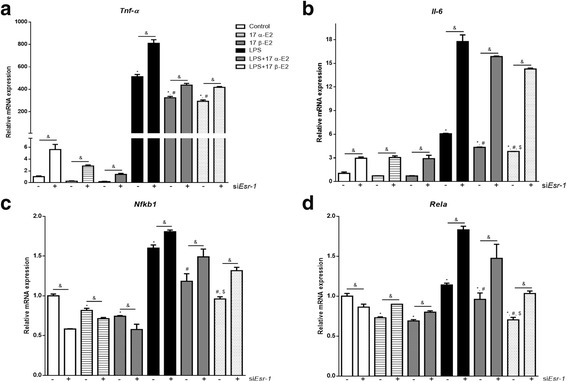
Effects of *Esr1* silencing on inflammatory markers gene expression in differentiated 3T3-L1 adipocytes. *Esr1* gene was silenced using siRNA, and cells were collected 72 h after silencing. Relative mRNA expression of *Tnf-α* (**a**), *Il-6* (**b**), *Nfκb1* (**c**), and *Rela* (**d**). Data are presented as mean ± SEM values. Asterisk indicates *P* < 0.05 compared to control group (C). Pound indicates *P* < 0.05 × LPS group. Dollar sign indicates *P* < 0.05 × LPS + 17 α-E2 group. Ampersand indicates *P* < 0.05 × siRNA group. (*n* = 3 independent rounds of cells)

Since the ERKO mouse lacks *Esr-1* gene expression in all tissues from the beginning of its embryonic development, we repeated our study by selectively knocking down *Esr-1* from fully differentiated adipocytes. Fully differentiated 3T3-L1 adipocytes were transfected with specific siRNA for *Esr-1*, which reduced *Esr-1* mRNA expression and protein (Additional file 2: Figure S2). Consistent with our hypothesis, knocking down *Esr1* resulted in higher levels of *Tnf-α* and *Il-6* in all groups. Furthermore, following knockdown of ERα, E2s were not able to decrease markers of inflammation to the same level (Fig. 6a–d).

### 17 α-E2 and 17 β-E2 attenuated LPS-induced inflammatory markers through decreased NFκB-p65 and increased ERα protein expression

**Fig. 7:**
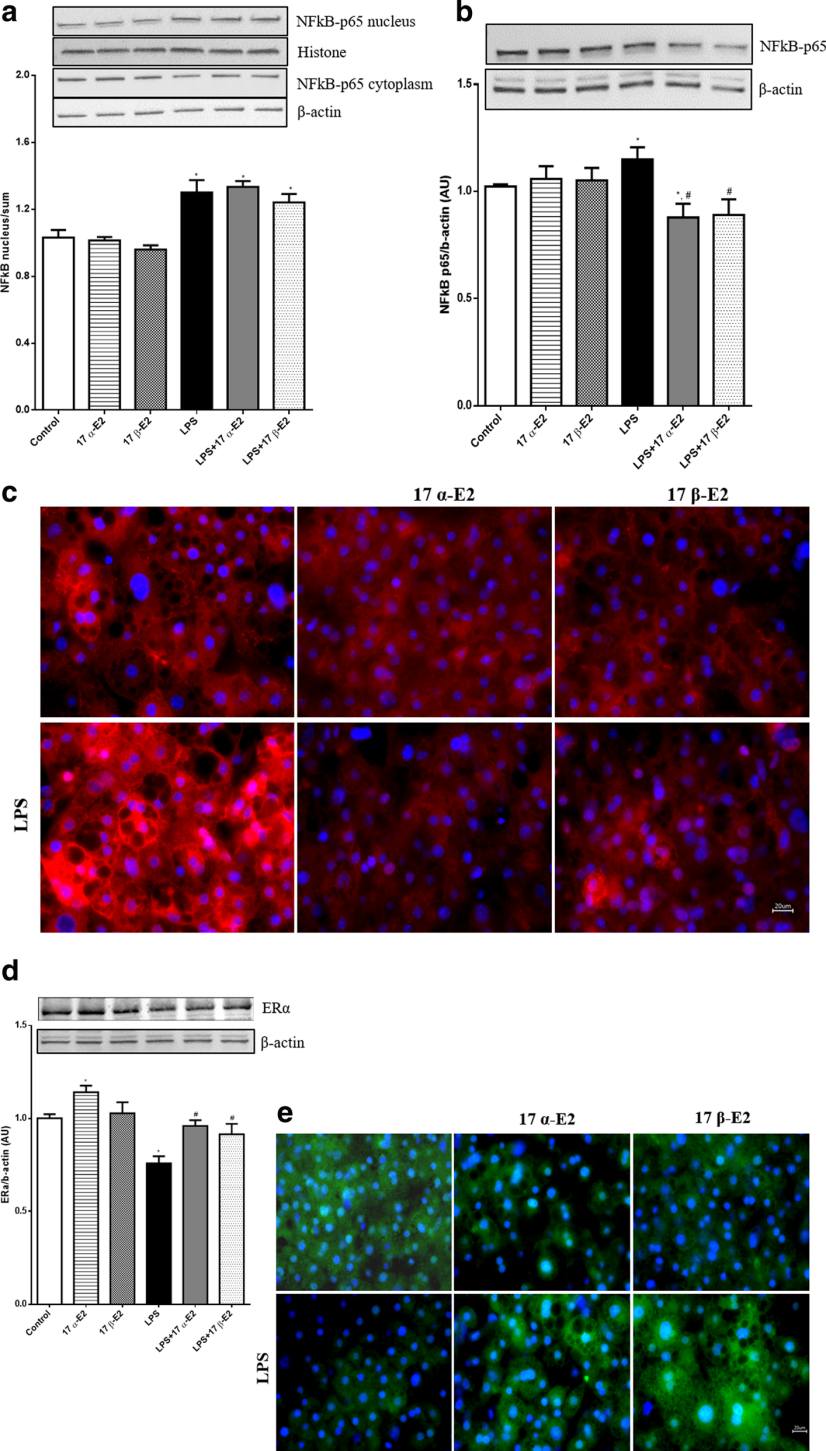
Effects of 17 α-E2 and 17 β-E2 on p65 and ERα protein expression in 3T3-L1 differentiated cells. **a** Nuclear and cytoplasmic fractions were extracted for Western blotting (WB) detection of p65. **b** Total protein was extracted for WB detection of p65. **c** Microscopy images of immunofluorescence labeling of p65 (red) in 3T3-L1 cells. Nuclei were stained with DAPI. **d** Total protein was extracted for WB detection of ERα. **e** Microscopy images of immunofluorescence labeling of ERα (green) in 3T3-L1 cells. Nuclei were stained with DAPI. Data are presented as mean ± SEM values. Asterisk indicates *P* < 0.05 compared to control group (C). Pound indicates *P* < 0.05 × LPS group. (*n* = 4 independent rounds of cells for WB, and *n* = 3 independent rounds of cells for immunofluorescence)

When stimulated, NFκB-p65 translocates to the nucleus and initiates the transcription of pro-inflammatory genes. To begin to investigate the mechanisms involved in E2-mediated reductions in inflammation, nuclear and cytoplasmic p65 protein levels were determined. Consistent with previous reports, LPS increased nuclear translocation of p65; however, here, we provide data suggesting *neither* isoform of E2 influenced translocation (Fig. 7a). Importantly, however, we did find that total protein levels of p65 were suppressed by both E2s (Fig. 7b), which was further demonstrated by immunofluorescence (Fig. 7c). Interestingly, LPS decreased ERα protein expression, whereas both estrogens prevented LPS-mediated decreases in ERα protein expression (Fig. 7d, e).

## Discussion

There is epidemiological evidence suggesting that estrogens protect against the development of chronic inflammatory diseases. This is further supported by the fact that following menopause, when circulating estrogens diminish, there is a concomitant increase in pro-inflammatory cytokines [8]. 17 Beta-estradiol (17 β-E2) has been well established as one of the estrogens that is responsible for mediating these anti-inflammatory effects [38–40], and this may be therapeutically important because both systemic inflammation, as well as inflammation within adipose tissues, mediates many of the comorbidities associated with obesity [3, 4, 14]. Critically, there are data demonstrating that 17 β-E2 decreases inflammation directly within adipose tissues [41, 42].

17 Alpha-estradiol (17 α-E2) is a natural, non-feminizing isomer of 17 β-E2 [15, 20], and recently, there have been reports that 17 α-E2 reduces inflammation in aged male mice [20]. Therefore, since 17 α-E2 is non-feminizing, it is important to determine if this could be used as an anti-inflammatory agent that would provide benefit without causing the negative/secondary effects of estrogen supplementation, such as uterine or breast cancer [27–30]. Here we extend our knowledge of the role of 17 α-E2 in modulating inflammation by directly looking at its impact in adipocyte-like cells. Our data support previous findings with respect to 17 α-E2’s ability to suppress inflammation within these tissues [20]. Additionally, we found that there may be a sexually dimorphic effect of 17 α-E2, with males being more sensitive to its effects.

We and others have reported an important role for ERα in modulating inflammatory responses in various tissues [9, 42]. Additionally, we have previously demonstrated that ERα is the predominant ER in the adipose tissue, and there are studies suggesting that ERα polymorphisms lead to adipose tissue accumulation, increased insulin resistance, and inflammation [10]. When we knocked down ERα expression in 3T3-L1 adipocytes, levels of inflammatory markers significantly increased. These data suggest the ability of 17 α-E2 to decrease markers of inflammation depends on ERα. The importance of ERα was further substantiated by our finding that LPS both suppresses the expression of ERα and induces inflammation, suggesting that one of the mechanisms by which inflammation is induced by LPS is through suppression of ERα. Importantly, it appears there is a sexual dimorphism regarding which ER mediates 17 α-E2’s effects on inflammatory markers. In contrast to what appears to be true for female cells, in males, there may be a different mechanism as it appears that ERs other than ERα may modulate 17 α-E2’s effects, such as ERβ and GPER30 (Additional file 3: Figure S3 and Additional file 4: Figure S4).

We have not specifically tested competitive binding of the ERs following exposure to differing E2s; however, reports indicate that 17 α-E2 and 17 β-E2 possess different affinities for ERs. According to Edwards and McGuire (1980) [43], there are variations in 17 α-E2’s relative binding affinity to ERs that could range from as low as 1% of 17 β-E2 in rat uterus to values reaching 90% in mouse uterus. Other investigators have found diverse affinities, as well: Kuiper et al. (1997) [44] reported an affinity of 17 α-E2 to ERα of 58% of the relative affinity of 17 β-E2, and 11% to ERβ, while Torand-Allerand et al. (2005) [26] reported an affinity of 17 α-E2 binding to human recombinant ERα and ERβ of 51 and 64% compared to 17 β-E2, respectively. Kaur et al. (2015) [45] indicated an affinity of 17 α-E2 to ERα to be 40-times lower than 17 β-E2. These reports suggest that in an environment where both E2s are present (as is the case in humans and mice in vivo), competition for ER binding may occur; however, since 17 β-E2 has higher binding affinity to both ERs, it tends to exert more biological activity. We suggest there may be physiological benefit in applying 17 α-E2 to reduce inflammatory markers when the levels of 17 β-E2 are reduced, such as in males and postmenopausal (post- oophorectomy) females.

NFkappaB (NFκB) is included within in the inflammatory cascade. Its inactive form resides in the cytoplasm, bound to IKappaB (IκB). When it is activated by a stimulus, IκB is phosphorylated by IKappaK (IκK) and dissociates from NFκB. NFκB then heterodimerizes and translocates to the nucleus, where it binds and modulates expression of target genes such as Tnf-α and Il-6. Estrogen receptors (ERs) can repress NFκB activity through diverse mechanisms. In the study of Quaedackers et al. (2001) [46], the osteoblastic cell line U2-OS was transfected with a NFκB reporter in combination with an expression vector encoding ERα or ERβ. Co-transfection with ERα resulted in repression of the TNFα-induced transcriptional activity of NFκB; however, co-transfection of ERβ resulted in upregulation of TNFα-induced NFκB activity in the absence of a ligand, leading to upregulation of NFκB-regulated genes, which suggests that the hormone-independent ER repression of NFκB is receptor-specific to ERα. Sun et al. (1998) [47] transfected HeLa-ER^+^ cells with a construct containing the IL-6 promoter linked to the luciferase gene NFκB p65 or NFκB p50 with IκBα or control expression vector. The transfection of p50 or p65 vectors alone induced IL-6 promoter activation. However, treatment of cells with estrogens (E2) abolished the IL-6 promoter activity, which was similar to the transfection of p50 and p65 with IκBα construct, which completely abrogated the NFκB-mediated IL-6 promoter activity. When cells were stimulated with phorbol ester (PMA), the levels of cytosolic IκBα protein were hugely decreased, while when co-treated with E2 and PMB, the PMA-induced IκBα protein degradation was inhibited. One possible explanation is that E2 stabilizes IκBα protein by interfering with its phosphorylation or ubiquination, inhibiting its proteolysis. There was no data in this study regarding which ER was transfected to HeLa cells that was mediating E2’s effects.

In the review of Kalaitzidis and Gilmore (2005) [48], the authors discuss that ERα, in an estrogen-dependent manner, inhibits NFκB activity in various cell lines, including Hep2, MCF-7, U2-OS, HeLa, F9, 293, and U937, and that this can occur by various mechanisms. One of the mechanisms they suggest is direct binding of ERα to p50/p52 and RelA/c-Rel heterodimers in the nucleus, which in turn blocks the binding of NFκB to the promoter region of target genes, such as the IL-6 promoter, preventing their transcription. Moreover, E2 binding to ERα can affect IκB processing possibly through E2-mediated inhibition of IκK, which prevents IkB phosphorylation, degradation, and NFκB translocation to the nucleus. ERα can also affect the ability of NFκB to interact with coactivators, either by direct competition or through disruption of the ability of NFκB to bind with coactivators, including Bcl-3, p300, and CBP among others.

The concentration of E2 is important for its ability to modulate NFκB; according to the literature, E2 concentrations equal to or above 100 pM are necessary to inhibit NFκB activation [8]. Additionally, in vitro studies have demonstrated that high concentrations of 17 β-E2 block LPS-induced NFκB-p65 DNA binding and transcriptional activity by preventing its nuclear translocation [49, 50]. This is important because in our study, we used an elevated concentration (10 μM) of both E2s, but they failed to prevent the translocation of the p65 subunit of Nfκb to the nucleus. We attribute the differences to differing cell types, as well as the timing and dosage of LPS/E2 treatments. Although E2s could not prevent the translocation of p65 to the nucleus, we did observe a reduction in p65 protein levels in cells, suggesting that reductions in the amount of p65 protein available for nuclear translocation may underlie the reduced transcription of pro-inflammatory genes.

It is important to note that the objective of the experiments described here was to directly compare the same concentrations of the different E2s in order to discern differences in their ability to modulate inflammatory markers. The concentration used in these studies was equivalent to the pharmacological dose of 17 β-E2 used in vivo [51, 52]. Currently, the literature is unclear as to whether the concentrations of these estrogenic hormones differ, and more specifically, if they differ by tissue type. Future studies are important to determine circulating and tissue specific levels of these hormones, as well as to test physiological levels of each estrogen to begin to characterize their similarities and differences. This will be extremely important to be done for both sexes, as we predict the tissue levels of these estrogens may differ by sex. This is an emerging field. While we believe we have begun to understand how these hormones function to reduce inflammation, more work is required to mechanistically understand their function at the tissue level.

## Conclusions

In conclusion, we provide new evidence demonstrating an anti-inflammatory role for 17 α-E2 in adipocytes, which appears to require ERα in females, but not in males. Furthermore, our data suggest a potential mechanism by which 17 α-E2 provides an anti-inflammatory effect which is by reducing NFκB p65 subunit transcription and translation, which, in turn, decreases transcription of inflammatory markers such as *Tnf-α* and *Il-6*. These findings are important because there is increasing interest in attaining the clinical benefits of estrogen compounds while avoiding adverse side effects. Since 17 α-E2 is a non-feminizing estrogen, its use may not cause the potential deleterious effects of feminizing estrogens. As such, our study suggests that this estrogen may be pharmacologically relevant for treatment of inflammatory diseases in males, as well as in postmenopausal females.

## Additional files

Additional file 1: Figure S1. Effects of 17 α-E2 and 17 β-E2 on modulation of viability of cells and lipolysis in 3T3-L1 differentiated cells. (**A**) Cell viability in % after estrogens and LPS treatments. (**B**) Oil red O staining for lipolysis in all groups. Data were presented in mean ± SEM values. (TIFF 2622 kb)

Additional file 2: Figure S2. Effects of *Esr1* silencing on *Esr-1* gene and ERa protein expression in 3T3-L1 differentiated cells. (**A**) Relative mRNA expression of *Esr1*. (**B**) Total protein was extracted for Western blotting detection of ERα after 72 h of siRNA. Data were presented in mean ± SEM values. Symbol (*) indicates *P* < 0.05 compared to control group (C). (&) indicates *P* < 0.05 x siRNA group. (*n* = 3 independent rounds of cells). (TIFF 375 kb)

Additional file 3: Figure S3. mRNA expression of *Esr2* (**A**) and *Gper1* (**B**) in primary pre-adipocyte cells derived from male mice. Data were presented in mean ± SEM values. Symbol (&) indicates *P* < 0.05 compared to WT mice. (*n* = 3 independent rounds of cells). (TIFF 375 kb)

Additional file 4: Figure S4. mRNA expression of *Esr2* (**A**) and *Gper1* (**B**) in Mef cells derived from WT and ERKO male mice. Data were presented in mean ± SEM values. Symbol (&) indicates *P* < 0.05 compared to WT mice. (*n* = 3 independent rounds of cells). (TIFF 239 kb)
