# Supplementary figures and images for: The effects of 17 alpha-estradiol to inhibit inflammation in vitro
Source: Biol Sex Differ. 2017 Sep 6;8:30. doi: 10.1186/s13293-017-0151-9 (PMC5586055; doi:10.1186/s13293-017-0151-9)

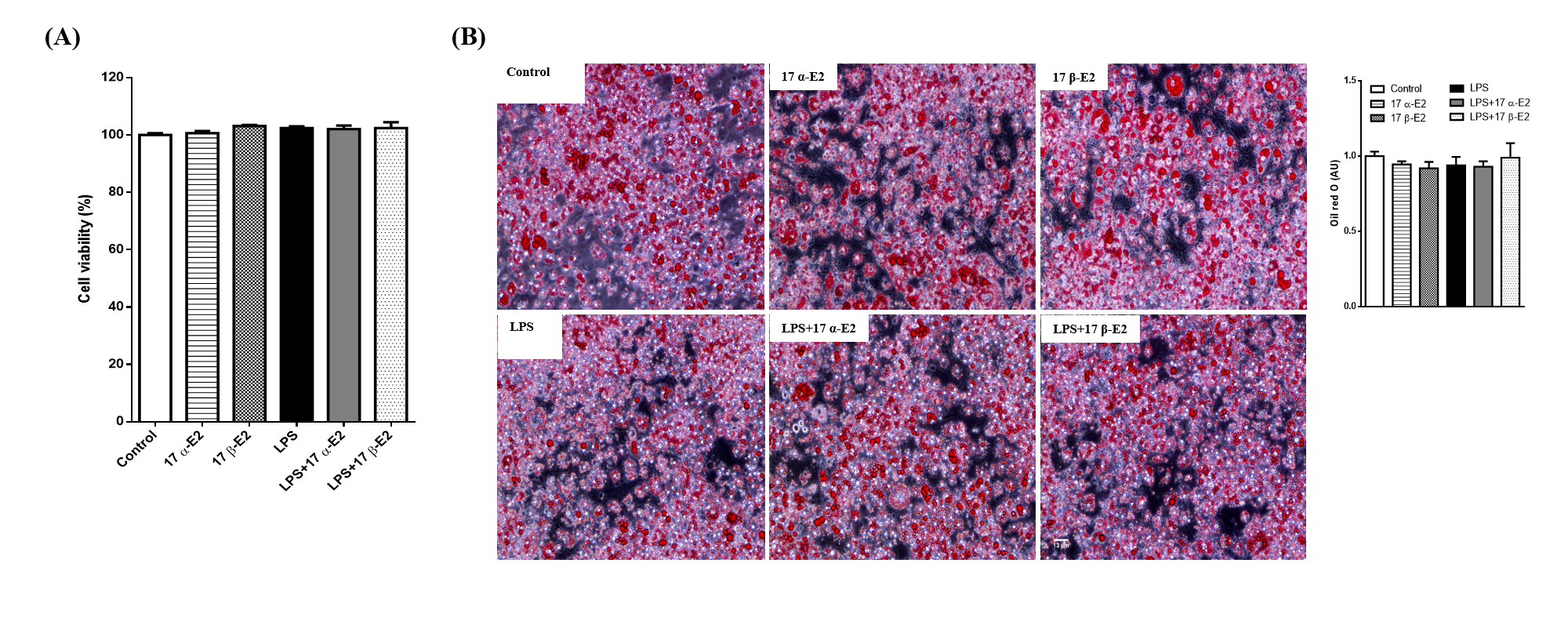

Supplement: Supplementary file 1 — Effects of 17 α-E2 and 17 β-E2 on modulation of viability of cells and lipolysis in 3T3-L1 differentiated cells. (A) Cell viability in % after estrogens and LPS treatments. (B) Oil red O staining for lipolysis in all groups. Data were presented in mean ± SEM values. (TIFF 2622 kb) [file 13293_2017_151_MOESM1_ESM.tif]

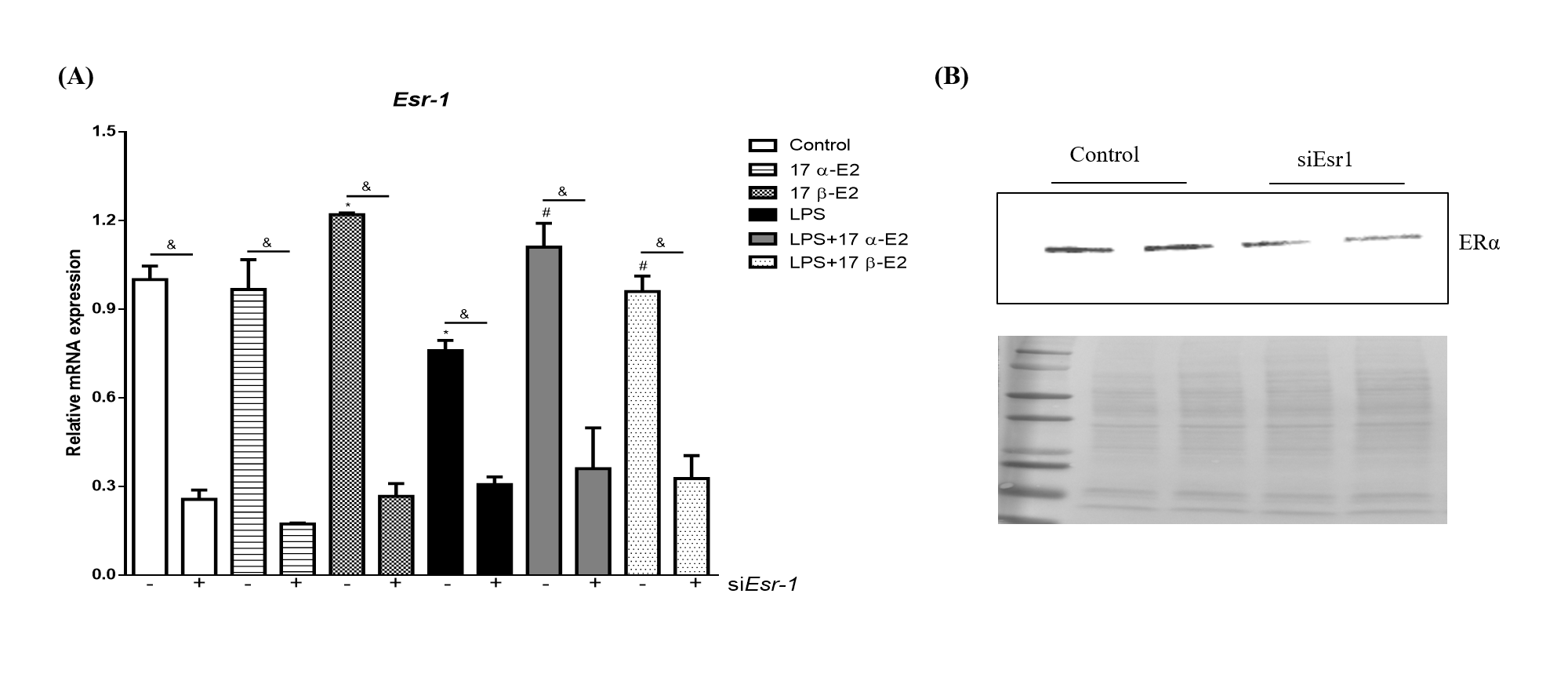

Supplement: Supplementary file 2 — Effects of Esr1 silencing on Esr-1 gene and ERa protein expression in 3T3-L1 differentiated cells. (A) Relative mRNA expression of Esr1. (B) Total protein was extracted for Western blotting detection of ERα after 72 h of siRNA. Data were presented in mean ± SEM values. Symbol (*) indicates P < 0.05 compared to control group (C). (&) indicates P < 0.05 x siRNA group. (n = 3 independent rounds of cells). (TIFF 375 kb) [file 13293_2017_151_MOESM2_ESM.tif]

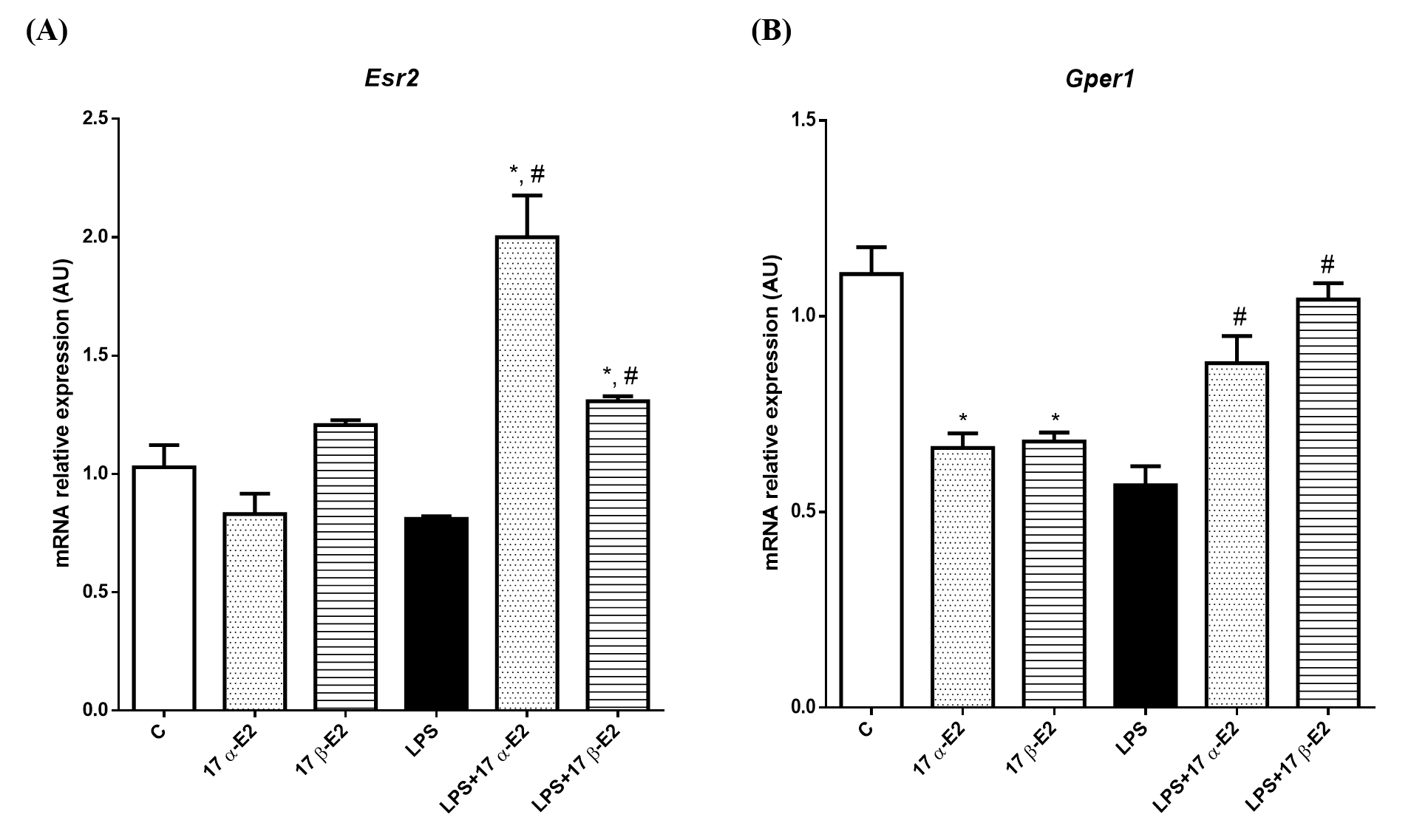

Supplement: Supplementary file 3 — mRNA expression of Esr2 (A) and Gper1 (B) in primary pre-adipocyte cells derived from male mice. Data were presented in mean ± SEM values. Symbol (&) indicates P < 0.05 compared to WT mice. (n = 3 independent rounds of cells). (TIFF 375 kb) [file 13293_2017_151_MOESM3_ESM.tif]

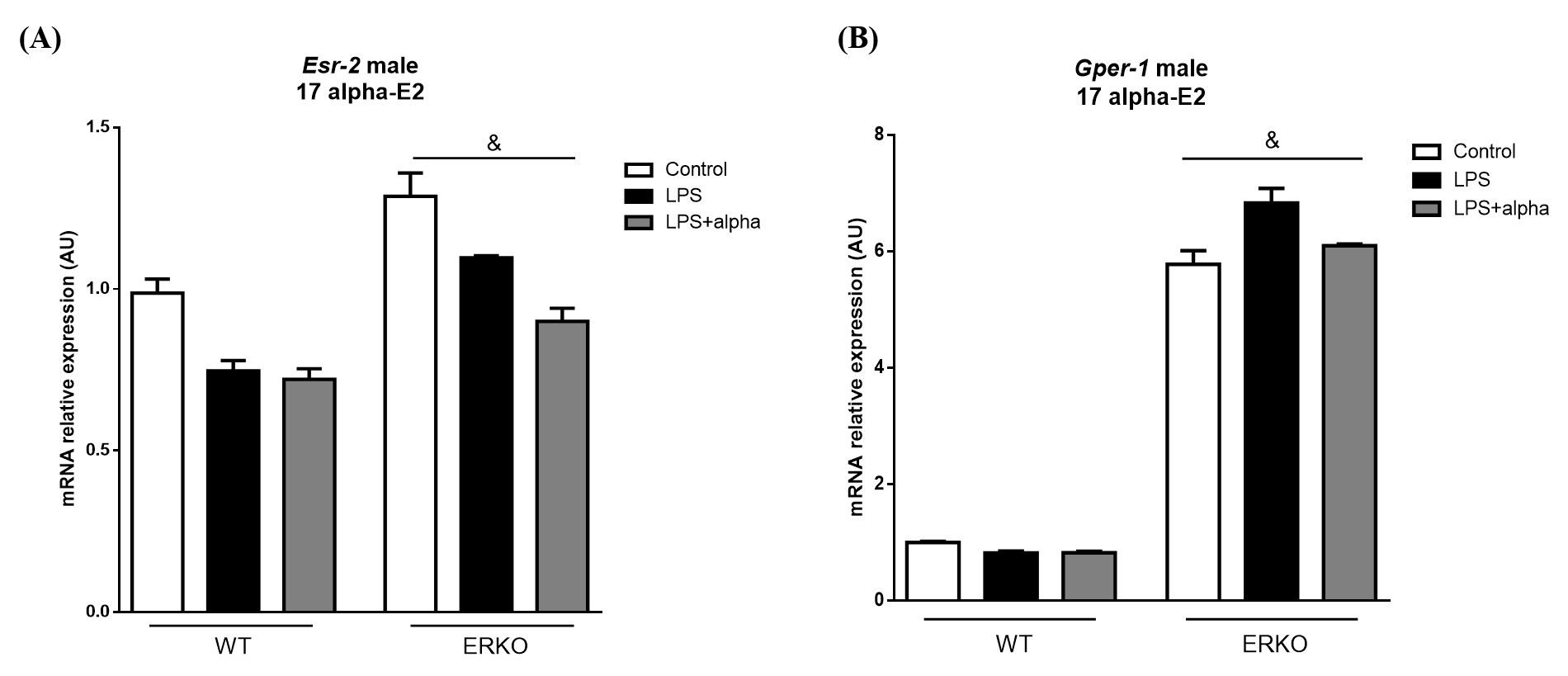

Supplement: Supplementary file 4 — mRNA expression of Esr2 (A) and Gper1 (B) in Mef cells derived from WT and ERKO male mice. Data were presented in mean ± SEM values. Symbol (&) indicates P < 0.05 compared to WT mice. (n = 3 independent rounds of cells). (TIFF 239 kb) [file 13293_2017_151_MOESM4_ESM.tif]
